# Human Impacts Flatten Rainforest-Savanna Gradient and Reduce Adaptive Diversity in a Rainforest Bird

**DOI:** 10.1371/journal.pone.0013088

**Published:** 2010-09-30

**Authors:** Adam H. Freedman, Wolfgang Buermann, Edward T. A. Mitchard, Ruth S. DeFries, Thomas B. Smith

**Affiliations:** 1 Center for Tropical Research, Institute of the Environment, University of California Los Angeles, Los Angeles, California, United States of America; 2 Department of Ecology and Evolutionary Biology, University of California Los Angeles, Los Angeles, California, United States of America; 3 Department of Atmospheric and Oceanic Sciences, University of California Los Angeles, Los Angeles, California, United States of America; 4 School of Geosciences, University of Edinburgh, Edinburgh, United Kingdom; 5 Department of Ecology, Evolution, and Environmental Biology, Columbia University, New York, New York, United States of America; Duke University, United States of America

## Abstract

Ecological gradients have long been recognized as important regions for diversification and speciation. However, little attention has been paid to the evolutionary consequences or conservation implications of human activities that fundamentally change the environmental features of such gradients. Here we show that recent deforestation in West Africa has homogenized the rainforest-savanna gradient, causing a loss of adaptive phenotypic diversity in a common rainforest bird, the little greenbul (*Andropadus virens*). Previously, this species was shown to exhibit morphological and song divergence along this gradient in Central Africa. Using satellite-based estimates of forest cover, recent morphological data, and historical data from museum specimens collected prior to widespread deforestation, we show that the gradient has become shallower in West Africa and that *A. virens* populations there have lost morphological variation in traits important to fitness. In contrast, we find no loss of morphological variation in Central Africa where there has been less deforestation and gradients have remained more intact. While rainforest deforestation is a leading cause of species extinction, the potential of deforestation to flatten gradients and inhibit rainforest diversification has not been previously recognized. More deforestation will likely lead to further flattening of the gradient and loss of diversity, and may limit the ability of species to persist under future environmental conditions.

## Introduction

Recent empirical [1]–[5] and theoretical [6]–[8] research suggests divergent selection along ecological gradients is an important evolutionary force in diversification and speciation. Fundamental to these assertions is that the strength of natural selection leading to reproductive isolation and speciation is related to the steepness of the environmental gradient [6], [8], where steepness refers to the rate of environmental turnover per unit geographic distance. Generally, steeper ecological gradients should translate into stronger differential selection capable of overcoming the homogenizing effects of gene flow and therefore be more likely to lead to diversification and speciation [6], [9]. In contrast, the flattening of ecological gradients, such as that caused by human landscape modification, may weaken the strength of divergent selection. Flattening occurs when habitats along gradients become progressively homogenized, and as a result, spatial turnover in environmental conditions is reduced. This may tip the balance in favor of the homogenizing effects of gene flow, undermining the potential for divergence [9].

It is increasingly recognized that anthropogenic environmental change can lead to rapid phenotypic evolution on the order of decades or less, across a taxonomically broad array of species [10]–[12]. Amongst these perturbations, habitat degradation and fragmentation are well-documented generators of novel selection pressures [10], [12]. The ability of local populations to persist in the face of habitat alteration depends critically upon the amount of heritable genetic variation in traits involved in local adaptation [10], [13]. Lacking genotypes favorable under the new conditions, a population's fitness will decrease, and its phenotypic shift to accommodate the new conditions will be slow—potentially too slow for it to persist. The increased risk of extinction due to a failure to adequately track anthropogenic environmental change has been termed “winnowing” [12]. Recent evidence in birds [14], [15] and reptiles [16] indicates that deforestation leads to rapid phenotypic evolution. If deforestation alters the distribution of adaptive variation along ecological gradients important to the generation and maintenance of intra-specific diversity, it could eventually constrain future evolutionary responses to new environmental conditions.

In equatorial Africa, the ecological gradient between rainforest and savanna has been shown to be important for the generation of rainforest biodiversity, particularly that portion of the gradient that spans rainforest and the rainforest-savanna transition zone (hereafter referred to as “ecotone”) [1], [4], [17]–[19]. As a result, human alterations of this gradient may have significant evolutionary consequences. African rainforests are under substantial human pressure [20], and deforestation resulting from logging and road construction is predicted to increase [21], [22]. The ecotone is a vast mosaic of gallery forests and wooded savannas, within which the abundance of woody vegetation is principally determined by anthropogenic disturbance [23]. However, because the magnitude of temporal change in woody vegetation within the ecotone is much smaller relative to that caused by deforestation in rainforest, we focus on how rainforest loss influences the slope of the rainforest-savanna gradient. We use newly available data on tree cover, derived from measurements from the Moderate Resolution Imaging Spectrometer (MODIS) sensors onboard NASA's Terra and Aqua satellites, to quantify the steepness of the gradient across West and Central Africa. We also use a time series of tree cover data based on measurements from the Advanced Very High Resolution Radiometer (AVHRR) sensor onboard NOAA's satellite fleet to examine recent trends that may be indicative of ongoing deforestation.

Greater deforestation in West Africa relative to Central Africa [24], [25] provides a comparative framework for assessing the evolutionary implications of changes to the rainforest-savanna gradient for species that inhabit both regions. In regions with high levels of deforestation, the slope of the rainforest-savanna gradient should be less steep. As a result, if morphological traits in a species are influenced by tree cover, such deforestation is predicted to lead to a reduction of divergence across the rainforest-savanna gradient. To examine how the flattening of this gradient may have impacted phenotypic diversity, we contrasted morphological divergence along the rainforest-savanna gradient between West and Central Africa in the sedentary bird species *Andropadus virens*, a species common in both rainforest and ecotone habitats [1]. Previous research on African passerine birds suggests that natural selection across the rainforest-savanna gradient has generated divergence in morphological and behavioral traits important in fitness and potentially speciation [1], [4], [17]–[19], [26], with *A. virens* exhibiting divergence in both morphology [1] and song [4] across the gradient. Previously, within the rainforest zone, *A. virens* has been shown to exhibit morphological, song and plumage differences in relation to tree cover [14]. We combined remote sensing and morphological analyses to: 1) quantify anthropogenic changes to the rainforest-savanna gradient in West Africa relative to Central Africa, 2) quantify a broad, trans-regional relationship between tree cover–an important component of the gradient–and morphology for a species showing evolutionary divergence along the gradient, and 3) test the hypothesis that flattening of the gradient in West Africa has led to a loss of morphological divergence among populations distributed along it.

## Results

### Deforestation impacts on gradient

**Figure 1:**
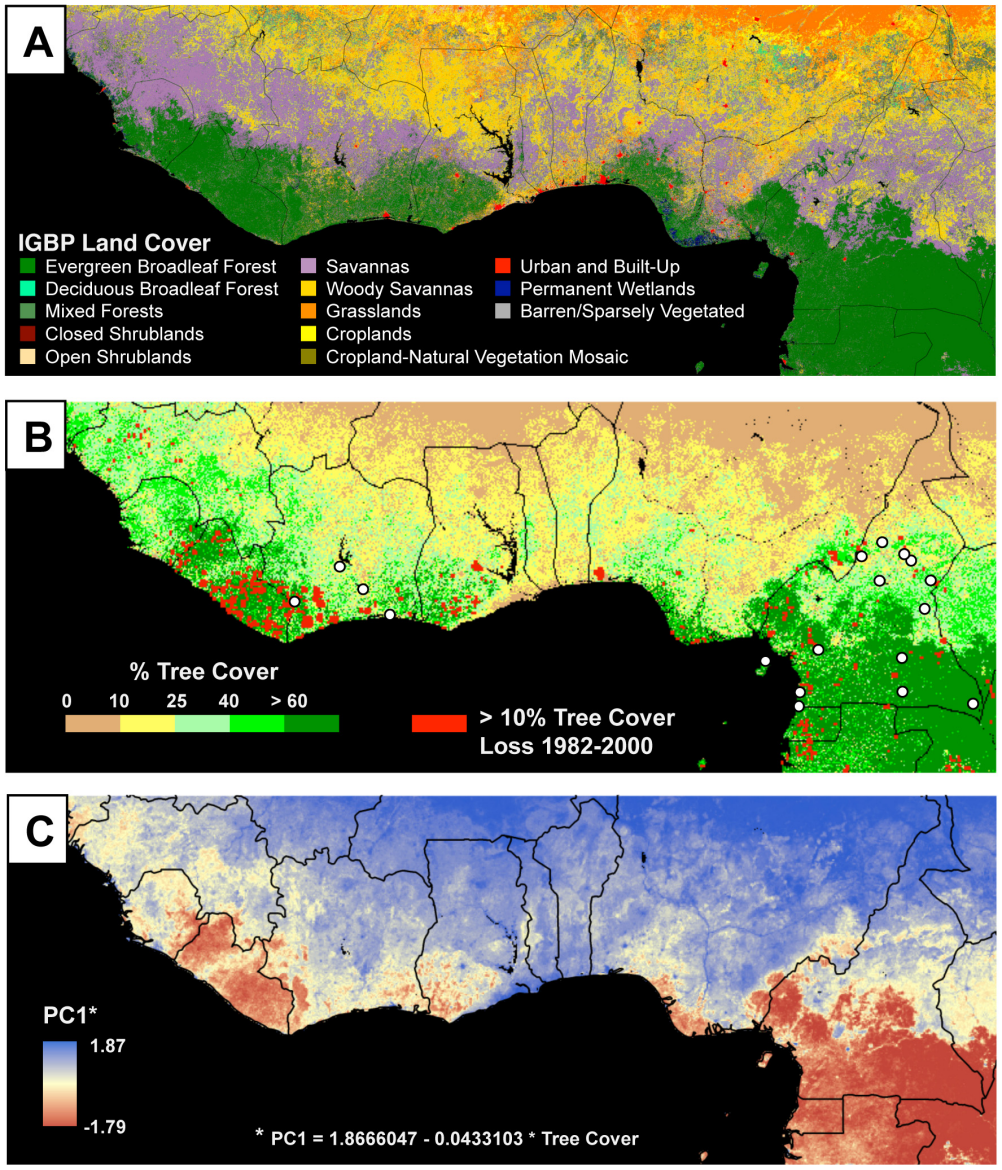
Potential and actual rainforest distribution, study sites, and consequences of gradient flattening for morphological diversity. (A) Land cover map based upon MODIS IGBP classification at 1 km spatial resolution for 2000 [28]. This classification includes information on vegetation cover and seasonality, and defines areas that, despite the extent of current deforestation, encompass suitable ecological conditions for rainforest. Areas classified as forest are broadly consistent with White's reconstruction of historical rainforest distribution [27]. (B) Present rainforest based on MODIS percent tree cover from 2001 [29], and location of study sites in West and Central Africa (indicated with circles). For sampling information, see Table S2. Sampling in West Africa coincides with a region where rainforest should occur, but where large losses of tree cover have led to convergence in habitat structure between the rainforest and ecotone zones. Red pixels indicate areas of recent deforestation (1982–2000), based upon tree cover data from measurements by the AVHRR satellite sensor [30]. Recent deforestation and gradient flattening have been greater in West than in Central Africa. (C) Projected distribution of PC1 (overall body size) of *A. virens*, estimated from a linear regression of PC1 on tree cover. This projection shows that in West Africa, other than in Sierra Leone, there is very little rainforest-ecotone divergence in PC1, and that deforestation in West Africa has led to the flattening of the morphological cline along the rainforest-savanna gradient. In contrast, in Central Africa, which has experienced much less deforestation, morphological divergence along the rainforest-savanna gradient persists. The same pattern is apparent for other traits in *A. virens* associated with tree cover (Figure S1).

**Figure 2:**
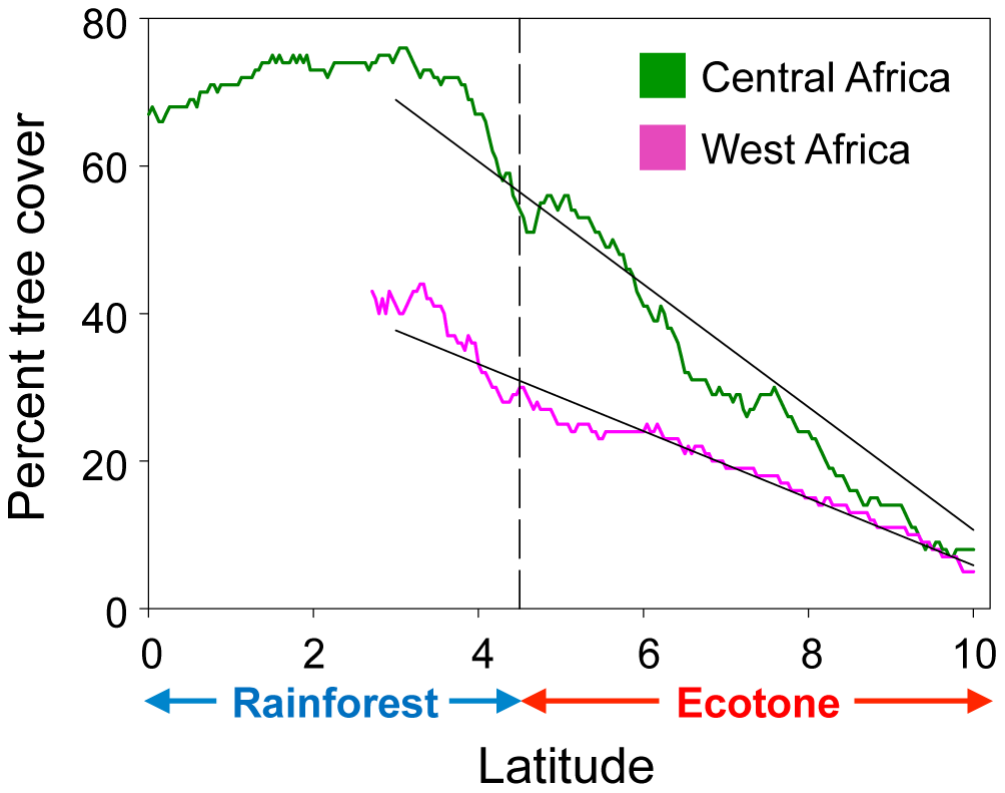
Greater flattening of the rainforest-savanna gradient in West than Central Africa. Rainforest-savanna gradients are represented by MODIS percentage tree cover plotted against latitude for West and Central Africa. For each region, mean tree cover was calculated for each 5km latitudinal band and plotted along the south-north direction. For easier comparison, the gradient for West Africa is shifted southward by 2.5° to account for the shift in latitudinal position of the rainforest-ecotone boundary between West and Central Africa. See Text S1 for details. The dashed line indicates the approximate position of the boundary between rainforest and the rainforest-savanna ecotone, and the solid black lines indicate least-square regression lines fit to the regional trends in tree cover-by-latitude trends.

In regions where rainforest should have occurred historically [27], [28], the spatial distribution of tree cover detected by the MODIS satellite sensors [29] indicates that West Africa has lost much more of its forest than Central Africa (Figure 1). The extent of human impacts on forest cover have been so extensive that many regions of West Africa today have tree cover comparable to, or in some cases, lower than ecotone habitats (Figure 1B). In the regions where we collected morphological data, this spatial pattern in forest cover results in a significantly shallower rainforest-savanna gradient in West (Ivory Coast) relative to Central (Cameroon and Equatorial Guinea) Africa (ANCOVA, Region * Latitude effect on percent tree cover *F* = 345.2, *p*<0.0001; Figure 2). The magnitude of differences in levels of rainforest tree cover and gradient slope between the two regions are the net result of human rainforest removal on a multi-decadal timescale. Coarse-scale AVHRR-based trends in tree cover from 1982 to 2000 provide a temporal perspective, and confirm the larger losses of tree cover in West Africa (Figure 1B) [30].

### Contemporary morphological diversity

**Figure 3:**
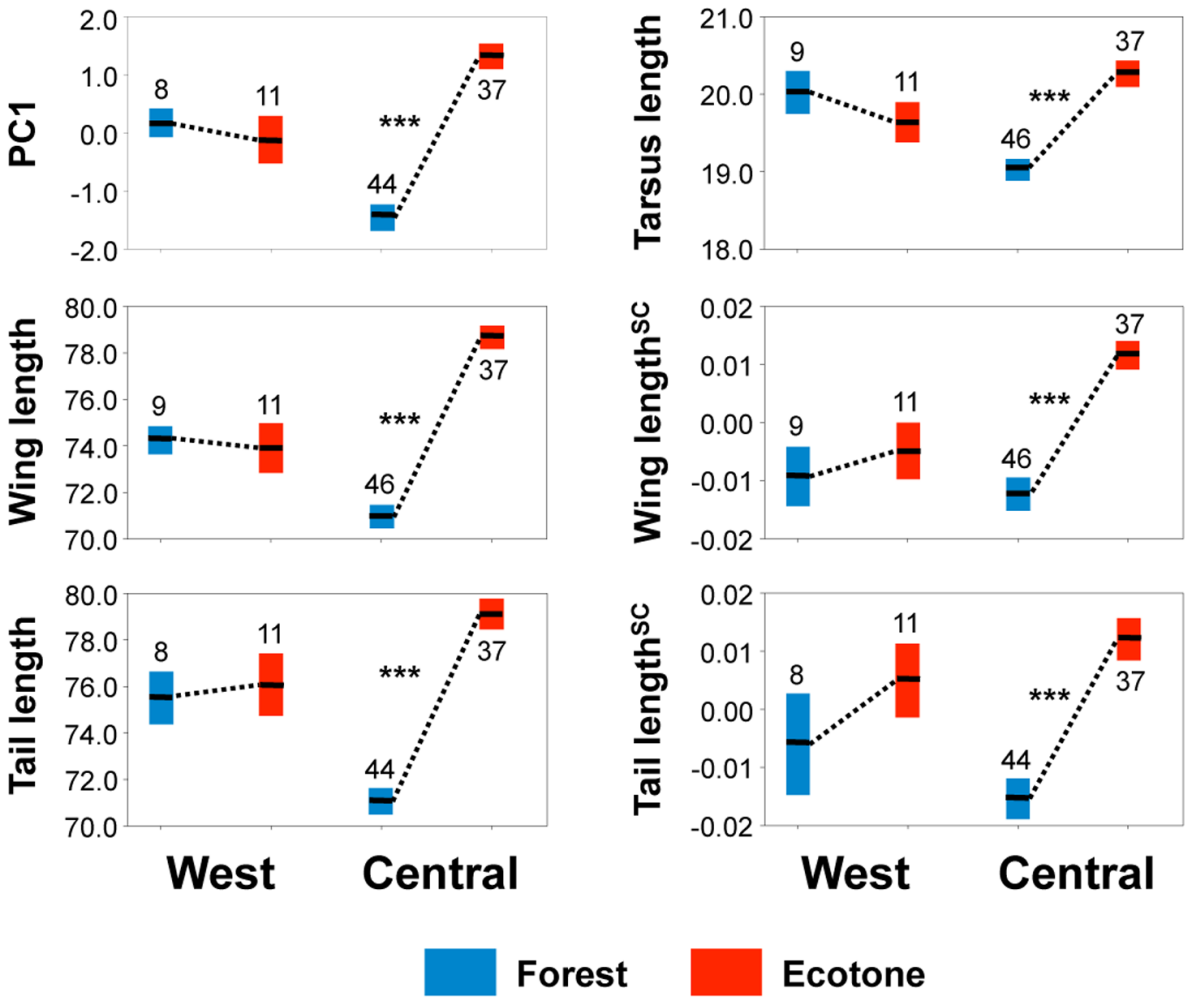
Morphological differences between rainforest and ecotone populations of *A. virens* in West and Central Africa. Morphological differences between rainforest and ecotone populations are smaller in West Africa than in Central Africa consistent with a flatter environmental gradient. Error bars indicate ±1 SE about the mean; sample sizes are adjacent to error bars. PC 1 is based upon tarsus, wing, tail, and upper mandible length. Size-corrected traits (indicated with SC superscript) are computed from regressions of log-transformed measurements on log-transformed tarsus length. Measurements of individual traits are in millimeters. Significant Wilcoxon rank sum tests (*p*≤0.001) comparing rainforest and ecotone habitats within regions are indicated by ***. Sample sizes and results of regressions of morphological traits on tree cover are provided in Table S1.

Overall size as indexed by PC1, tarsus, and both absolute and size-corrected wing and tail length, were significantly associated with percent tree cover (Table S1). These significant associations were all in the direction expected from observed patterns of rainforest-ecotone divergence in Central Africa (Figure 3), with birds becoming more ecotone-like in morphology as forest cover decreases.

Projections based on regressions of morphological traits on tree cover show that deforestation has led to a severe flattening of the cline in morphology along the gradient in West Africa, with the exception of Sierra Leone, where considerable forest remains (Figures 1C and S1). We detected no morphological divergence along the rainforest-savanna gradient in West African populations (Figure 3). In contrast, in Central Africa, where there has been less deforestation the gradient is steeper and morphology is significantly divergent along the gradient in PC1, tarsus length, and both absolute and size-corrected wing and tail length (Figure 3). Further, bootstrap resampling shows that the lack of morphological differentiation in West Africa relative to Central Africa is extremely unlikely to have been an artifact of sampling (PC1, *p*<0.001; absolute and size-corrected wing length, *p*<0.001; tail length, *p*<0.0001; size-corrected tail length, *p* = 0.013; tarsus length, *p*<0.001). Population-level re-sampling yielded similar results (see Text S1), consistent with a lack of morphological divergence in West Africa.

Inter-regional differences in gene flow between rainforest and ecotone populations are unlikely to explain the observed lack of morphological divergence in West Africa. First, pairwise F_ST_ between habitats is actually higher in West Africa than in Central Africa (West Africa, F_ST_ = 0.058±0.005 SE, range = 0.053–0.063, *n* = 2; Central Africa, F_ST_ = 0.013±0.001 SE, range = 0.0001–0.024, *n* = 30), with the the largest F_ST_ observed in Central Africa is less than half of that observed in West Africa. The opposite would be true if the homogenizing effect of gene flow was contributing to the observed lack of morphological divergence among West African populations. Second, even if our limited sampling in West Africa has substantially underestimated rainforest-ecotone gene flow, the level of gene flow required to entirely homogenize morphology seems unrealistic in light of previous research on *A. virens*. Among rainforest-ecotone population comparisons in Cameroon, several-fold increases in gene flow did not reduce rainforest-ecotone divergence to that observed between pairs of rainforest populations [1]. Therefore, our results suggest the loss of morphological divergence between habitats depends upon the extent of tree cover along the gradient, and that the loss of morphological diversity in traits typically shown to be important in fitness in birds [31] has been greater in West Africa where deforestation has been more extensive [24], [25], [30].

### Pre-deforestation morphological diversity

**Figure 4:**
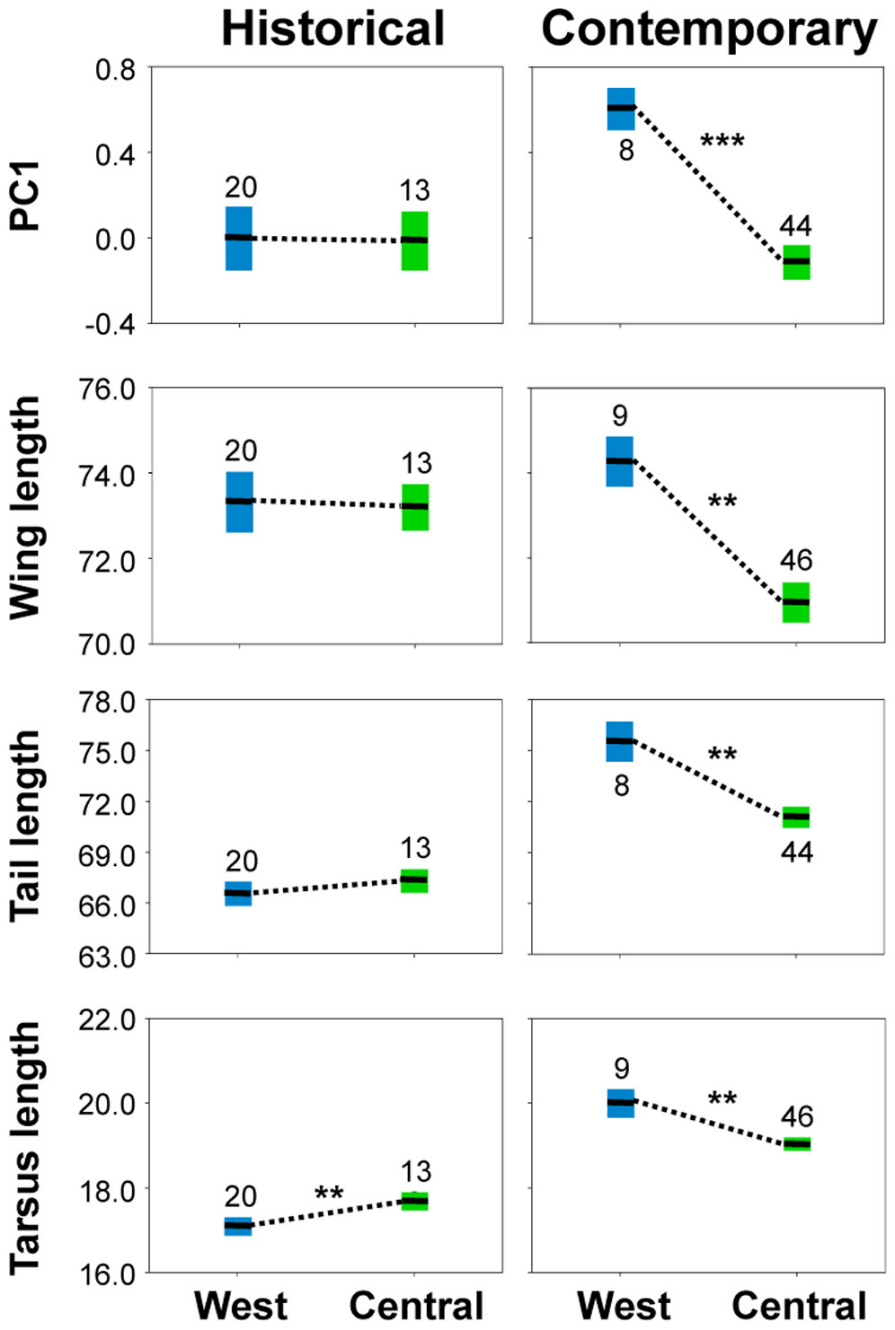
Historical and contemporary morphological differences between West and Central Africa rainforest populations of *A. virens*. Regional populations are more morphologically divergent now than they were historically, due to West African populations becoming more ecotone-like as a result of deforestation. Error bars indicate ±1 SE about the mean; sample sizes area adjacent to error bars. PC1 is based upon tarsus, wing, tail, and upper mandible length, and to facilitate visualization are normalized, within time period, by the largest individual value so that PCs scale from −1 to +1. Measurements of individual traits are in millimeters. Significant Wilcoxon rank sums tests comparing regions within each time interval are indicated by ** and ***, denoting *p*≤0.01 and *p*≤0.001, respectively.

To provide an independent test of gradient flattening and loss of morphological diversity, we measured museum specimens of *A. virens* from the late 1800's and early 1900's, before large-scale deforestation of the region following World War II [32]. Consistent with the high levels of deforestation in West Africa, rainforest populations from West and Central Africa are morphologically more divergent today than they were historically (Figure 4). This was true for all traits we examined. A shift in West Africa towards more ecotone-like morphology is likely responsible for the increase in divergence between regions, and occurs in all traits in which we detected significant rainforest-ecotone divergence in Central Africa. Furthermore, the shift is consistent with the direction of morphological change observed in *A. virens* when humans clear rainforest [14]. Thus, temporal changes in morphology over the last 100 years in West Africa appear to be tracking the conversion of rainforest to more open canopy, ecotone-like habitats.

## Discussion

The flattening of the rainforest-savanna gradient caused by human destruction of rainforest appears to be erasing a signature of divergence-with-gene-flow [33], a pattern previously described for *A. virens*, and thought to be important in speciation [1], [4], [17], [34]. Should land use practices in Central Africa continue on their present trajectory [21], flattening of the gradient is likely to lead to losses of phenotypic diversity similar to that observed in West Africa. This prediction is supported by both the inverse relationship between morphological traits and tree cover and previous research on this species showing morphological shifts resulting from deforestation [14].

Depending upon the dispersal potential of species, the loss of adaptive diversity along the gradient could be the product of the dispersal of individuals from ecotone habitats into previously forested ones, *in situ* evolution via natural selection in human-altered rainforest habitats or a combination of both. However, in the case of *A. virens*, its sedentary habits [1], and rapid evolutionary response to forest alteration at fine spatial scales within the rainforest zone [14], suggest that microevolutionary forces are likely driving divergence. While the phenotypic intermediacy of forest and ecotone populations in West Africa relative to their Central African counterparts (Figure 3) could suggest a greater role in West Africa for the homogenizing effect of gene flow, the greater neutral genetic differentiation between habitats in West than Central Africa suggests this is unlikely. Less gene flow along the rainforest-savanna gradient in West than in Central Africa is opposite to what would be expected if the spatial proximity of forest and ecotone sites in West Africa was responsible for the observed morphological homogeneity there. Even if gene flow along the gradient in West Africa was underestimated, empirical estimates of divergence-with-gene-flow among *A. virens* populations in Cameroon suggest unrealistically high levels of genetic exchange would be necessary to produce the absence of morphological divergence observed in West Africa.

While the lack of museum specimens from ecotone habitat limited us to contrasting historical and contemporary variation in rainforest populations across regions, analyses for both time periods were performed for the same pair of regions. As a result, they would likely share any unaccounted for effects, making the relative levels of differentiation between them informative. In contrast, comparing contemporary morphological traits among both habitats and regions does not allow one to avoid possible confounding regional differences (e.g., intrinsic differences in species riches between regions that could influence morphology through competition). Absent information regarding historical inter-regional differences in ecotone morphology, any conclusions regarding the magnitude of gene flow from rainforest into ecotone populations seem speculative, especially in light of relatively large amounts of gene flow that would be necessary to erase morphological divergence between habitats.

Finally, although sampling was restricted to a smaller number of field sites in West Africa, population and individual-level resampling analyses demonstrate that the lack of rainforest-ecotone divergence in West Africa could not solely be due to low statistical power arising from smaller sample sizes or asymmetric sampling of populations. Regardless of whether the lack of rainforest-ecotone divergence in West Africa is due to the replacement of rainforest individuals with dispersers from ecotone (unlikely due to the greater genetic differentiation between habitats there), or whether *in situ* phenotypic evolution has occurred in response to deforestation, there is a reduction of variation in traits important to fitness where the gradient is less steep.

Previous research on the evolutionary consequences of deforestation for rainforest birds has shown that fluctuating assymetry in tarsus length increases in response to deforestation, and a negative relationship between fluctuating assymetry and species' persistence in rainforest fragments [35], [36]. However, other than our previous work on *A. virens* documenting the phenotypic responses to deforestation at small spatial scales [14], we are unaware of other studies showing directional change in rainforest birds in response to deforestation. We find that a suite of traits may respond to loss of forest cover, including not only tarsus length, but those related to flight performance as well. Generally, longer wings are predicted to be better adapted for more open habitats while shorter, rounder wings are favored in more closed habitats [37], [38]. Similar to our findings, a negative relationship was found between tree cover and wing length in a South American rainforest bird [39]. The larger wings characteristic of ecotone birds may reflect the need to fly more rapidly in open grassland between gallery forests to avoid predators.

Recent studies have documented phenotype-tree cover relationships for other African vertebrates, suggesting that deforestation may produce evolutionary responses in other taxa. In an African rainforest lizard, morphological traits and genes under selection vary along the gradient [40]. In addition, phenotypic, genetic, and song divergence along this gradient has been detected in other bird species besides *A. virens* [17], [19], [26], [41]. These studies along with those on other continents [15], [16] suggest that evolutionary change generated by rainforest deforestation could be a common phenomenon. Further work will be required to determine the extent to which gradient flattening may be impeding diversification generally or even reversing speciation [42]. But given the documented associations between tree cover and phenotype in other species, the observed and potential further truncation of morphological diversity in *A. virens* in response to gradient flattening may be a proxy for a broader pattern of reduced genetic variation in fitness-related phenotypic traits for other taxa distributed across the gradient.

Perhaps more important than the reduction of future diversification relative to historical baselines, gradient flattening may impose evolutionary limits upon species' ability to persist in the face of future environmental change, whether due to continuing habitat alteration, invasive species, global warming, or some other novel, unanticipated perturbation. In the context of climate change, standing genetic variation has proven sufficient for adaptive responses in some species [43], but not in others [44]. Little is known about levels of variation in functionally important traits across species, or about the level of variation necessary for adaptation to future environmental regimes. However, given uncertain knowledge of future environments and the fact that the Africa continent is predicted to be more affected by climate change than any other [45], conservation strategies should strive to maximize adaptive phenotypic variation wherever possible.

While there has been much focus on population declines and extinctions of rainforest species as a result of deforestation [46], our results suggest that the threats to ecological gradients and the important evolutionary processes that promote diversity along them need greater attention [47], [48]. We provide the first evidence linking loss of an evolutionarily significant environmental gradient with a documented decline in morphological diversity. However, the ecological signatures associated with deforestation suggest a larger global-scale phenomenon. In West Africa, deforestation and gradient flattening have been associated with the loss of rainforest species and incursions by savanna-adapted species into previously forested regions [49], [50], and such incursions arising from deforestation have also recently been documented in Cameroon [51], [52]. Similar changes associated with rainforest deforestation have also been documented in Southeast Asia [53] and South America [54], [55]. Within South America, the flattening of environmental gradients in the Andes appears to be particularly acute [56], [57]. To preserve both the pattern of biodiversity and the processes that produce and maintain it, preservation of gradients should be considered as an important component of conservation strategies complementary to more traditional prioritization approaches [48], [58]. With as much as two thirds of the world's terrestrial land area impacted by human activities [59] gradient flattening is likely widespread, but its corresponding impacts on adaptive diversity and implications for conservation in a changing world are not fully recognized.

## Materials and Methods

### Ethics statement

Research was conducted under San Francisco State University's and UCLA's Institutional Animal Care and Use Committee (IACUC) protocols.

### Field sampling

Field sampling was conducted at 17 sites in West Africa (Ivory Coast) and Central Africa (Equatorial Guinea and Cameroon) between 1993 and 2001 (Figure 1B; Table S2). Sites were classified as either (a) forest, including primary and secondary lowland rainforest [1], [18], [60], [61], (b) ecotone, comprised of mosaics of savanna, forest patches, and gallery forest [1], [18], [60]–[62], or (c) montane [63], [64]. These classifications were determined from our field observations of habitat, the expected distribution of rainforest in Africa derived from continent-wide field surveys [27], and from the International Geosphere-Biosphere Programme landcover (IGBP) map generated from data collected by the MODIS satellite sensor [28]. The IGBP landcover classification is generated from remotely sensed environmental features such as live vegetation, surface texture, and temperature that collectively describe the *potential* for an area to support rainforest vegetation. This potential can be juxtaposed against the extent of tree cover, also estimated from the MODIS sensors, to indicate deforested sites in the rainforest zone. Thus, we classified sampling locations in West Africa as forest sites if the IGBP map classified them as forest, even if the level of tree cover was comparable to (or lower than) that found in ecotone sites. Mist netting protocols, morphological measurements, and DNA sample storage follow Smith et al. [18]. All morphological measurements were taken by TBS. To avoid any potential confounding effects of sexual dimorphism, we restricted our analyses to adult males (*n* = 126) for which sample sizes were larger than for females. Juveniles were distinguished from adults based on plumage characters [65], and sex determinations made in the field were verified using a PCR protocol that identifies a gene on the W chromosome [66].

### AVHRR-based NDVI trend analysis for detecting recent deforestation

To illustrate large-scale patterns of deforestation, we used an 18-year AVHRR record from the Pathfinder program (1982–2000) recorded by NOAA/NASA satellites [67], [68] at 8 km resolution. Areas where tree cover decreased more than 10% during this time period, computed from the AVHRR time series by DeFries et al. [30], [69]–[71], were overlaid onto the MODIS tree cover map.

### Tree cover gradient analysis for West and Central Africa

We determined whether the slope of the gradient in tree cover was different between West and Central Africa, restricting our analyses to those countries where we conducted field studies on *A. virens*—Ivory Coast in West Africa, and Cameroon and Equatorial Guinea in Central Africa. Specifically, we tested the hypothesis that the slope of the rainforest-savanna tree cover gradient is shallower in West Africa than in Central Africa using an ANCOVA, with latitude and region as factors, and a latitude * region interaction term. We confirmed that the rainforests of West and Central Africa would have been broadly comparable prior to deforestation by comparing levels of tree cover in rainforest protected areas from both regions. See Text S1 for details.

### Analysis of contemporary morphological variation

For live-captured birds, we measured morphological traits typically important to fitness [31]: tarsus length, wing length, tail length, and upper mandible length. We also evaluated size-corrected traits, calculated as residuals from linear regressions of log-transformed traits on log-transformed tarsus length (a standard measure of body size). As feeding performance is a function of absolute bill morphology and food resources [31], [72], we did not correct upper mandible length measurements for body size. We performed principle components analysis (PCA) to calculate additional variables that describe overall size (PC 1) and shape (PC 2). Before performing PCA across geographic regions, we verified that region-specific covariance matrices were either equal or proportional [73] using the program CPC, available at http://www.uoregon.edu/~pphil/programs/cpc/cpc.htm.

To establish the relationship between morphology and tree cover, we performed univariate least-square weighted regressions of each PC or log-transformed trait on percent tree cover, with sites weighted by sample size. We included montane sites and a forest site on the island of Bioko in this analysis in order to take maximal advantage of sampling, and because we expected the relationship between morphology and tree cover to be general across habitats. Percent tree cover was collected from a 2001 tree cover map available at 500m resolution, constructed from measurements made by the MODIS satellite [29]. For each morphological trait that was significantly associated with tree cover, we generated a predictive map of its distribution by inputting the tree cover map into the corresponding regression equation, using the Spatial Analyst Raster Calculator in ArcGIS 9 [74]. These projections were then aggregated to 5 km resolution, and back-transformed for traits that were log-transformed for the regression analyses. See Text S1 for additional details.

To determine whether loss of morphological differentiation between rainforest and ecotone habitats was greater in West than in Central Africa, we analyzed variation in PC 1 and log-transformed morphological traits between rainforest and ecotone. We restricted our analyses to continental lowland rainforest and ecotone sites. Because the distribution of tarsus length within these habitats was non-normal even after log-transformation, and to accommodate for small sample sizes in some comparisons, we evaluated morphological divergence with Wilcoxon rank sum tests (2-tailed, α = 0.05). We excluded PC2 and upper mandible length from these and subsequent analyses, because these traits were not significantly associated with percent tree cover—the environmental feature with which we define the rainforest-savanna gradient (Table S1; additional methods in Text S1). We applied Dunn-Sidak correction [75] for multiple forest-ecotone comparisons, within regions, for the six traits showing a significant association with tree cover (α = 0.05; α' = 1−(1−α)1/6 = 0.0085).

Because sampling in West Africa was limited relative to Central Africa, we tested whether the lack of morphological divergence between habitats in West Africa could result from sampling unrepresentative individuals. For each morphological trait in which we found significant morphological divergence in Central but not West Africa, we bootstrapped 1,000 samples from the pool of individuals captured in Central Africa. These samples were drawn so that the numbers of individuals from rainforest and ecotone habitats were identical to those collected in West Africa. We then calculated the probability of observing a lack of rainforest-ecotone divergence, as the proportion of replicates where the absolute morphological divergence between rainforest and ecotone was less than or equal to that observed in West Africa. We also tested whether the lack of morphological divergence in West Africa was due to chance sampling of atypical populations (details in Text S1). Additionally, we confirmed that the lack of morphological divergence between rainforest and ecotone habitats in West Africa was not due to higher levels of between-habitat gene flow than in Central Africa, by calculating mean between-habitat F_ST_ from matrices provided in Smith et al. [18].

### Analysis of historical morphological variation

To confirm that regional differences in rainforest-ecotone morphological divergence were due to greater deforestation in West Africa, and not to a persistent historical pattern, we collected morphological data from historical specimens of *A. virens* deposited at the Natural History Museum at Tring, United Kingdom (Table S3, details in Text S1). As with live-captured birds, TBS took all measurements. To ensure that sampled birds were collected prior to extensive deforestation, we only included individuals collected before 1935 [32]. We also excluded individuals for which locality information was not available or did not permit an assignment to either rainforest or ecotone habitat with reasonable confidence. Locality and habitat information were obtained from historical records (e.g., accounts of particular collectors' expeditions published in *Ibis*), georeferencing tools such as online gazetteers and Google Earth [76], and contemporary tree cover from MODIS. Localities falling within areas of high contemporary tree cover were presumed to have been historically covered by rainforest.

Very few individuals from the museum collection that met our criteria for inclusion were from ecotone habitats. This is likely due to collecting bias during this time period, as many early collectors were either under the employ of resource extraction companies operating near the coast where forest predominates, or focused their collection efforts in coastal areas due to ease of access. We recomputed the PCA for contemporary samples excluding individuals from ecotone, montane, and island habitats, and performed a separate PCA on the historical samples. Although included in the analyses of contemporary rainforest-ecotone differentiation, we did not evaluate morphological divergence in size-corrected wing and tail measurements, because for museum specimens regressions of these traits on tarsus length were not statistically significant. We evaluated the significance of differences between regional rainforest populations with Wilcoxon rank sum tests as noted above. All statistical analyses were performed using JMP 7.0 [77], with the exception of bootstrapping, which was implemented with a Microsoft Excel spreadsheet macro.

## Supporting Information

Text S1 Additional detailed information concerning methods and results.
(0.04 MB DOC)

Table S1 Weighted least-squares regressions of morphological traits on percent tree cover.
(0.05 MB PDF)

Table S2 Coordinates, habitat classification, percent tree cover, and sample sizes for A. virens captured and measured by TBS in West and Central Africa.
(0.06 MB PDF)

Table S3 Historical specimens of A. virens measured at the Natural History Museum at Tring, United Kingdom.
(0.06 MB PDF)

Figure S1 Additional projections of morphological traits, based upon the associations between traits and tree cover. Associations are estimated with least-squares linear regression. For all traits, morphological diversity in West Africa has been lost due to flattening of the rainforest-savanna gradient.
(3.16 MB PDF)
