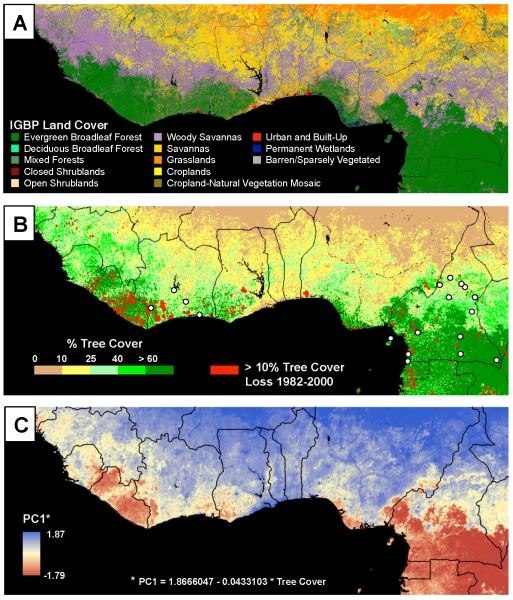# Correction: Human Impacts Flatten Rainforest-Savanna Gradient and Reduce Adaptive Diversity in a Rainforest Bird

**DOI:** 10.1371/annotation/d50ccb46-fd60-450d-8c2c-4c3be512deac

**Published:** 2010-10-29

**Authors:** Adam H. Freedman, Wolfgang Buermann, Edward T. A. Mitchard, Ruth S. DeFries, Thomas B. Smith

Figure 1 was published with the labels for Savannas and Woody Savannas reversed in part A. Please view the correct figure 1 here: 

**Figure:**