# Supplementary material for: Human Impacts Flatten Rainforest-Savanna Gradient and Reduce Adaptive Diversity in a Rainforest Bird
Source: PLoS One. 2010 Sep 30;5(9):e13088. doi: 10.1371/journal.pone.0013088 (PMC2948002; doi:10.1371/journal.pone.0013088)
Supplement: Text S1 — Additional detailed information concerning methods and results. (0.04 MB DOC) [file pone.0013088.s001.doc]

**Supporting Text**

**Title:** Human Impacts Flatten Rainforest-Savanna Gradient and Reduce Adaptive Diversity

**Authors:** Adam H. Freedman^1,2^,*, Wolfgang Buermann^1,3^, Edward T.A. Mitchard^4^,

Ruth S. DeFries^5^, and Thomas B. Smith^1,2^

^1^ Center for Tropical Research, Institute of the Environment, University of California Los Angeles, Los Angeles, California, United States of America

^2^ Department of Ecology and Evolutionary Biology, University of California Los Angeles, Los Angeles, California, United States of America

^3^ Department of Atmospheric and Oceanic Sciences, University of California Los Angeles, Los Angeles, California, United States of America

^4^ School of Geosciences, University of Edinburgh, Edinburgh, United Kingdom

^5^ Department of Ecology, Evolution, and Environmental Biology, Columbia University, New York, New York, United States of America

* Author for correspondence ([afreedma@ucla.edu](mailto:afreedma@ucla.edu))

**Materials and Methods**

**Tree cover gradient analysis for West and Central Africa**

We reaggregated the MODIS tree cover map to 5 km resolution. For each region, we then calculated mean tree cover for each latitudinal row with > 50 grid cells. Because the location of the rainforest-ecotone boundary differs in latitude between West and Central Africa, to make plots comparable we shifted the plot for West Africa by -2.5°. We determined the relative location of this boundary from the IGBP landcover map (figure 1a). We truncated the regional data sets so as to start at the latitude at which the maximum mean tree cover was located. This effectively excludes areas outside the relevant latitudinal range in Central Africa, and those areas containing natural inland savannas in Gabon. The longitudinal range for latitudinal averaging of tree cover was ≈7.53°W − 2.94°W for West Africa (Ivory Coast), and ≈ 8.60°E − 16.24°E for Central Africa (Cameroon, Equatorial Guinea, and Gabon).

In addition to evidence for historically large, contiguous blocks of rainforest in both Central and West Africa [S1,S2], we sought further confirmation that the density of rainforest in these regions prior to deforestation (and thus their associated rainforest-savanna gradients) were comparable with respect to vegetation structure. To do so, we compared the distribution of tree cover in two protected rainforest areas: Tai National Park in Ivory Coast, and the Dja Biosphere Reserve in Cameroon. As protected rainforest areas in West Africa, including Tai National Park, have been impacted by logging [e.g. S3], we expect *a priori* reduced tree cover in Tai National Park relative to the Dja Biosphere Reserve where logging has not occurred. Nevertheless, the magnitude of differences in tree cover, and the proportions of tree cover in the highest density classes can provide some indication of whether the rainforest-savanna gradient in West Africa was similiar in character to that in Central Africa, prior to the disproportionate, more recent impacts of deforestation.

Despite impacts of logging in Tai National Park, the difference in mean tree cover between the areas is modest (Tai National Park 64.05±0.12 SE, Dja Biosphere Reserve 77.06±0.06,Wilcox rank sum=103.9, *p*≤0.001). Means for both areas are above the minimum threshold that delineates continuous rainforest (≈60), and the majority of grid cells in both areas are above this threshold. Second, at the highest tree cover thresholds (>85%), the two protected areas contain similar proportions of rainforest. These findings support the potential for rainforest of comparable density in West and Central Africa, particularly in proximity to the study sites where we collected data on *A. virens* morphology.

**Analysis of contemporary morphological variation**

To account for georeferencing error, we used the average percent tree cover from a 2.5 x 2.5 km pixel centred on each study site. These tests are already conservative, because of georeferencing error, and because climate variables that may influence morphology are not correlated with the large amount of variation in tree cover within the rainforest zone due to human activity. As a result, a climate-morphology relationship would reduce our ability to detect a similar relationship with tree cover. Thus, we did not apply a correction factor to control for the familywise error rate arising from multiple individual tests. However, we point out that applying such a correction would only influence the significance of the regression of size-corrected wing length on tree cover. The normality of site means used in the regressions of each morphological trait were confirmed with Shapiro-Wilk W tests (all p>0.05).

**Analysis of historical morphological variation**

Although sex was obtained for most individuals, museum tags for eleven older historical specimens did not contain sex information, and lack of any distinctive plumage or other variation prevented us from classifying the sex of these individuals. We chose to include these individuals because doing so did not statistically influence our results except in the case of tarsus length, and this trait is not significantly sexually dimorphic in live-caught birds from Central African rainforest where we have large sample sizes and sufficient statistical power (Wilcoxon rank sum test on log-transformed tarsus length, male *n*=46, female *n*=40, χ^2^=0.0054, *p*=0.94).

**Population re-sampling analyses**

In addition to the bootstrapping analyses, we also calculated the probability that morphological divergence in West Africa was less than in Central Africa due to sampling atypical populations. Specifically, we calculated rainforest-ecotone divergence between all possible sets of two rainforest and two ecotone populations from Central Africa, with weighted means for each habitat calculated using the sample sizes of the corresponding populations in West Africa. For each trait, this produced 300 replicates. The probability of sampling populations lacking forest-ecotone divergence is extremely low for PC1 (*p*<0.001), absolute wing length (*p*<0.001), absolute tail length (*p*<0.001), and size-corrected wing length (*p*=0.013). For tarsus length and size-corrected wing length, 20% and 21% of replicates had a level of forest-ecotone divergence less than or equal to that observed in West Africa, respectively. However, for these two traits, the lack of forest-ecotone divergence was driven almost exclusively by one Central African population. For tarsus length, 96% of these replicates showing low divergence involved comparisons including Betare Oya. For size-corrected tail length, 88% of these replicates involved comparisons including Meiganga. Thus, for four of six traits, the lack of morphological divergence along the gradient cannot be explained by chance sampling of populations. And, because low divergence in tarsus length and size-corrected tail length were each driven by different populations, it is unlikely that those traits would simultaneously exhibit low divergence by chance alone. Multiplying their respective probabilities of low divergence, there is only a four percent chance that those traits would simultaneously show low forest-ecotone divergence. We can therefore rule out the effects of non-random sampling as the cause for the lack of morphological divergence in West Africa for five of six traits.

**References**

S1. White F (1983) Vegetation of Africa - a descriptive memoir to accompany the UNESCO/AETFAT/UNSO vegetation map of Africa. Natural resources report XX. Paris: UNESCO. 356p.

S2. Friedl MA, McIver DK, Hodges JCF, Zhang XY, Muchoney D, et al. (2002) Global land cover mapping from MODIS: algorithms and early results. Remote Sens Environ 83: 287−302.

S3. United National Environment Programme World Conservation Monitoring Centre (UNEP-WCMC) (2007) World Heritage sites, protected areas and World Heritage. Taï National Park, Côte d'Ivoire. Cambridge, United Kingdom: UNEP-WCMC. 9 p.
