# Supplementary material for: Human Impacts Flatten Rainforest-Savanna Gradient and Reduce Adaptive Diversity in a Rainforest Bird
Source: PLoS One. 2010 Sep 30;5(9):e13088. doi: 10.1371/journal.pone.0013088 (PMC2948002; doi:10.1371/journal.pone.0013088)
Supplement: Table S1 — Weighted least-squares regressions of morphological traits on percent tree cover. (0.05 MB PDF) [file pone.0013088.s002.pdf]

Table S1. Weighted least-squares regressions of morphological traits on percent tree cover.

Trait	r^2	+/- *	p[†]	n (observations)[‡]
PC1	0.51	–	**0.0009**	18 (125)
PC2	0.01		0.68	18 (125)
Tarsus length (mm)[§]	0.39	–	**0.006**	18 (128)
Wing length (mm)[¶]	0.48 (0.32)[‖]	–	**0.0015** (**0.015**)	18 (128)
Tail length (mm)[¶]	0.57 (0.45)[‖]	–	**0.0004** (**0.002**)	18 (125)
Upper mandible length (mm)[§]	0.04		0.41	18 (128)

* Indicates the slope of the regression, where significant, between morphology and tree cover.

[†] Boldface indicates significance.

[‡] Number of populations, with parentheses indicating the total number of individuals.

[§] Regressions of log-transformed trait values on log-transformed percent tree cover.

[¶] Regressions use residuals from linear regressions of log-transformed trait values on log-transformed tarsus length, which are then regressed on percent tree cover.

[‖] Parentheses indicate results for traits corrected for size using tarsus length.
