# Supplementary material for: Human Impacts Flatten Rainforest-Savanna Gradient and Reduce Adaptive Diversity in a Rainforest Bird
Source: PLoS One. 2010 Sep 30;5(9):e13088. doi: 10.1371/journal.pone.0013088 (PMC2948002; doi:10.1371/journal.pone.0013088)
Supplement: Table S2 — Coordinates, habitat classification, percent tree cover, and sample sizes for A. virens captured and measured by TBS in West and Central Africa. (0.06 MB PDF) [file pone.0013088.s003.pdf]

Table S2. Coordinates, habitat classification, percent tree cover, and sample sizes for *A. virens* captured and measured by TBS in West and Central Africa.

**Site**	**Latitude**	**Longitude**	**Country**	**Habitat**	**% Tree Cover**	***n***
1. Paule Oula	5.826°N	7.391°W	Ivory Coast	Forest	49.6	4
2. Lamto	6.216°N	5.027°W	Ivory Coast	Ecotone	19.7	9
3. Marahoue	7.028°N	5.948°W	Ivory Coast	Ecotone	24.9	2
4. CSRS	5.331°N	4.129°W	Ivory Coast	Forest	13.7	5
5. Bioko*	3.743°N	8.721°E	Equatorial Guinea	Forest	64.4	7
6. Elende	2.216°N	9.793°E	Equatorial Guinea	Forest	69.1	10
7. Kribi	2.731°N	9.872°E	Cameroon	Forest	77.7	4
8. Sakbayeme	4.038°N	10.574°E	Cameroon	Forest	56.5	16
9. Nkwouak	3.870°N	13.316°E	Cameroon	Forest	74.0	8
10. Zobefame	2.659°N	13.397°E	Cameroon	Forest	79.1	3
11. Lac Lobeke	2.311°N	15.762°E	Cameroon	Forest	79.5	5
12. Tchabal Mbabo*	7.252°N	12.058°E	Cameroon	Montane	33.6	4
13. Tchabal Gandaba*	7.743°N	12.716°E	Cameroon	Montane	30.9	14
14. Tibati	6.504°N	12.588°E	Cameroon	Ecotone	28.3	8
15. Wakwa	7.271°N	13.526°E	Cameroon	Ecotone	18.2	5
16. Ngaoundaba	7.133°N	13.698°E	Cameroon	Ecotone	28.0	5
17. Meiganga	6.517°N	14.300°E	Cameroon	Ecotone	17.6	6
18. Betare Oya	5.563°N	14.091°E	Cameroon	Ecotone	28.3	13

* Sites are used in weighted regressions of morphological traits on percent tree cover, but not for comparisons of morphological divergence between rainforest and ecotone habitats between West and Central Africa.
