# Supplementary material for: Human Impacts Flatten Rainforest-Savanna Gradient and Reduce Adaptive Diversity in a Rainforest Bird
Source: PLoS One. 2010 Sep 30;5(9):e13088. doi: 10.1371/journal.pone.0013088 (PMC2948002; doi:10.1371/journal.pone.0013088)
Supplement: Table S3 — Historical specimens of A. virens measured at the Natural History Museum at Tring, United Kingdom. (0.06 MB PDF) [file pone.0013088.s004.pdf]

Table S3. Historical specimens of *A. virens* measured at the Natural History Museum at Tring, United Kingdom.

Catalogue #	Year Collected	Locality	Country	Source
1902.7.15.5	1901	Efulen	Cameroon	1,2
1904.7.18.118	1904	Efulen	Cameroon	1,2
1905.1.24.131	1904	Dja River	Cameroon	1
1905.1.24.126	1904	Dja River	Cameroon	1
1906.12.14.93	1905	Efulen	Cameroon	1,2
1906.12.14.90	1905	Efulen	Cameroon	1,2
1906.12.14.94	1905	Efulen	Cameroon	1,2
1911.5.31.270	1905	Bitye	Cameroon	1
1911.5.31.271	1907	Bitye	Cameroon	1
1969.44.56	1907	Bitye	Cameroon	1
1920.6.26.406	1908	Assobam, Bumba River	Cameroon	3
1920.6.26.407	1908	Assobam, Bumba River	Cameroon	3
1924.11.2.137	1922	Bitye	Cameroon	1
1911.12.23.1632	1900	Prahsu	Gold Coast (Ghana)	4,5
1936.2.21.308	1900	Prahsu	Gold Coast (Ghana)	4,5
1911.11.18.124	1910	Sekondi	Gold Coast (Ghana)	4,6
1934.3.16.502	1934	Goaso, Ashanti	Gold Coast (Ghana)	4,7
1934.3.16.504	1934	Goaso, Ashanti	Gold Coast (Ghana)	4,7
1934.3.16.506	1934	Mampong, Ashanti	Gold Coast (Ghana)	4,7
1858.1.4.109	1858	Ashanti	Gold Coast (Ghana)	7
1876.5.23.606	1876	Fantee	Gold Coast (Ghana)	8,9
1876.5.23.643	1876	Fantee	Gold Coast (Ghana)	8,9
1876.5.23.646	1876	Fantee	Gold Coast (Ghana)	8,9
1876.5.23.338	1876	Fantee	Gold Coast (Ghana)	8,9
1876.5.23.645	1876	Fantee	Gold Coast (Ghana)	8,9
1876.5.23.604	1876	Fantee	Gold Coast (Ghana)	8,9
1895.5.1.718	1895	Fantee	Gold Coast (Ghana)	8,9
1895.5.1.715	1895	Fantee	Gold Coast (Ghana)	8,9
1895.5.1.714	1895	Denkera	Gold Coast (Ghana)	8,9
1911.11.18.126	1911	Nanna Kru	Liberia	6
99.8.10.27	1893	Grand Cape	Liberia	4
1930.12.3.290	1930	Kankordu	Sierra Leone	10
1930.12.3.289	1930	Sandaru	Sierra Leone	10

1 Louette, M (1981) The birds of Cameroon: an annotated checklist. Verhandl Kon Acad Wetensh Lett Schone Kunst Belg 43: 1–295.

2 Sharpe RB (1902) On a small collection of birds from Efulen in Cameroon, West Africa. Ibis 44: 89–96.

3 Ogilvie-Grant WR (1917) VI.—Remarks on some recent collections of birds made by Mr. G. L. Bates in Cameroon. Ibis 59: 72–90.

4 Online gazetteer.

5 Alexander B (1902) XIX.—On the birds of the Gold Coast colony and its hinterland. Ibis 44: 278–333.

6 Bannerman DA (1912) X.—On a collection of birds made by Willoughby P. Lowe on the west coast of Africa and outlying islands; with field-notes by the collector. Ibis 54: 219–268.
7 Lowe WP (1937) XVII.—Report on the Lowe-Waldron expeditions to the Ashanti forests and northern territories of the Gold Coast. Ibis 79: 345–368.
8 Sharpe RB (1869) On a Collection of Birds from the Fantee Country in Western Africa. Ibis 11: 186–195.
9 Sharpe RB, Ussher HT (1872) XXIII.—Three new Species of Birds from the Fantee Country. Ibis 14: 181–183.
10 Bannerman DA (1931) XLII.—Accounts of birds collected (i.) by Mr. G. L. Bates on behalf of the British Museum in Sierra Leone and French Guinea; (ii) by Lt.-Col. G. J. Houghton, R. A. M. C., in Sierra Leone, recently acquired by the British Museum. Ibis 73: 661–697.
