# Supplementary figures and images for: Human Impacts Flatten Rainforest-Savanna Gradient and Reduce Adaptive Diversity in a Rainforest Bird
Source: PLoS One. 2010 Sep 30;5(9):e13088. doi: 10.1371/journal.pone.0013088 (PMC2948002; doi:10.1371/journal.pone.0013088)

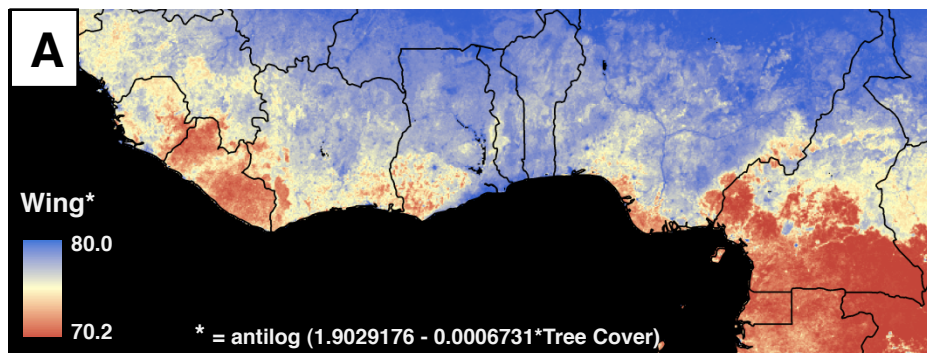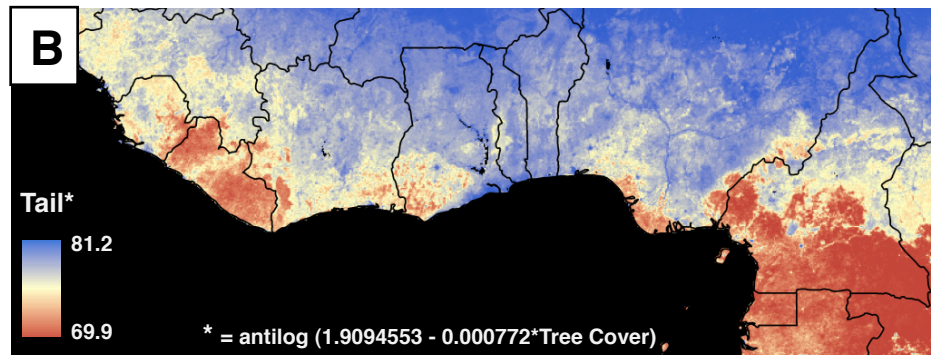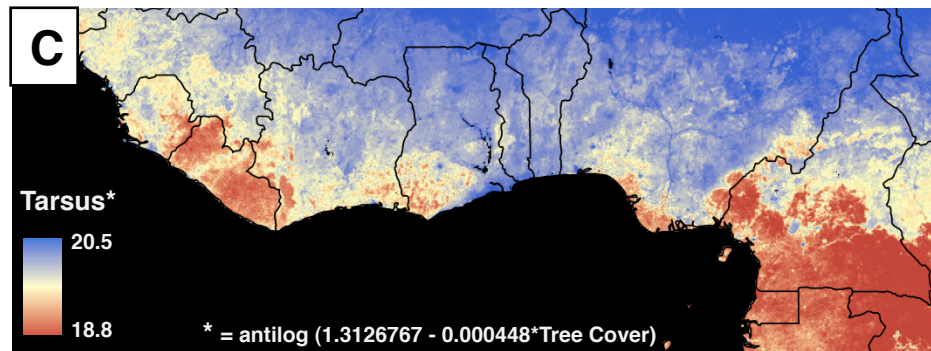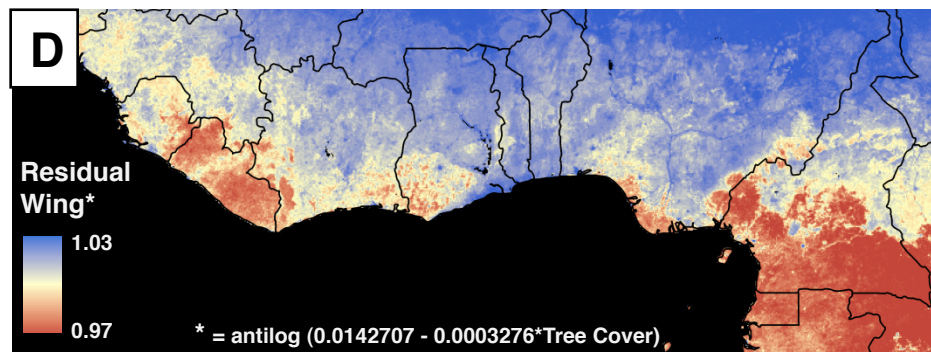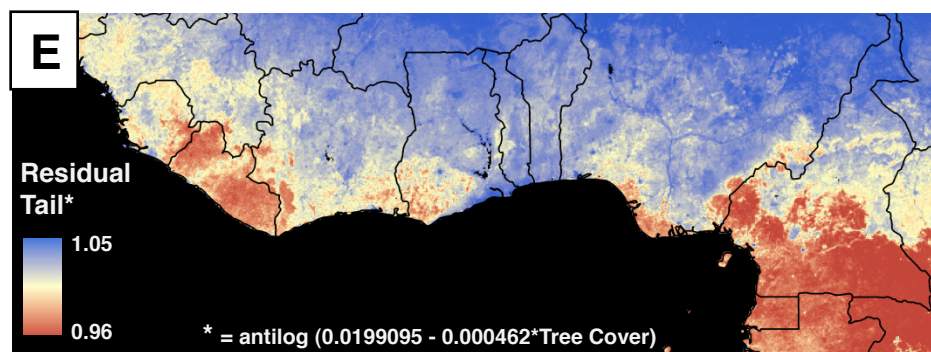

Supplement: Figure S1 — Additional projections of morphological traits, based upon the associations between traits and tree cover. Associations are estimated with least-squares linear regression. For all traits, morphological diversity in West Africa has been lost due to flattening of the rainforest-savanna gradient. (3.16 MB PDF) [file pone.0013088.s005.pdf]
